# Synthetic Ligands of Myeloid C‐Type Lectin Receptors

**DOI:** 10.1002/cbic.70449

**Published:** 2026-07-02

**Authors:** James Suri, Bernd Lepenies

**Affiliations:** ^1^ Chair of Biochemistry and Chemistry, Department of Veterinary Sciences, Faculty of Veterinary Medicine Ludwig‐Maximilians‐Universität München Planegg Germany

**Keywords:** C‐type lectin receptor, host**–**pathogen interactions, immunology, innate immunity, ligand design

## Abstract

C‐type lectin receptors (CLRs) are one of the major classes of pattern recognition receptors. They mediate numerous biological events, including cell adhesion, pathogen recognition, and innate immune responses. Initial recognition of a pathogen by specific CLRs shapes the inflammatory landscape of the host, and the resulting immune response can be protective but can also contribute to immune‐mediated pathology. Understanding the binding of CLRs to their ligands has gained increasing attention and triggered research in the fields of structural biology, pathogen recognition, and immune signaling. Of critical importance for understanding the factors that affect binding and, therefore, being able to therapeutically exploit CLRs as druggable targets, is the identification of binding epitopes and drug‐like analogs. Hence, informed tailoring of candidate molecules can enhance affinities, exploit avidity, and enable the design of novel ligands. This review surveys the extensive diversity that has been generated in the area of synthetic myeloid CLR ligands, focusing on the dendritic cell‐specific ICAM3‐grabbing non‐integrin receptor (DC‐SIGN), macrophage‐inducible C‐type lectin (Mincle), dendritic cell‐associated C‐type lectin‐1 (Dectin‐1), and langerin. Context is rendered via the description of binding modes, signaling pathways, and therapeutic opportunities, which facilitates the identification of current limitations and potential future directions of the field.

## Graphical abstract

Binding of ligands to C‐type lectin receptors (CLRs) on myeloid cells can lead to an array of cellular responses. Exploitation of these receptors for therapeutic purposes requires high‐affinity ligands. This review surveys the diversity of synthetic ligands of myeloid CLRs in the context of binding, signaling, and downstream effects, as well as potential therapeutic applications. Potential limitations and future directions are also highlighted.

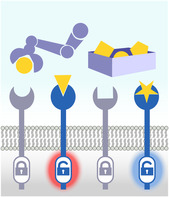

## 1 Introduction

**FIGURE 1:**
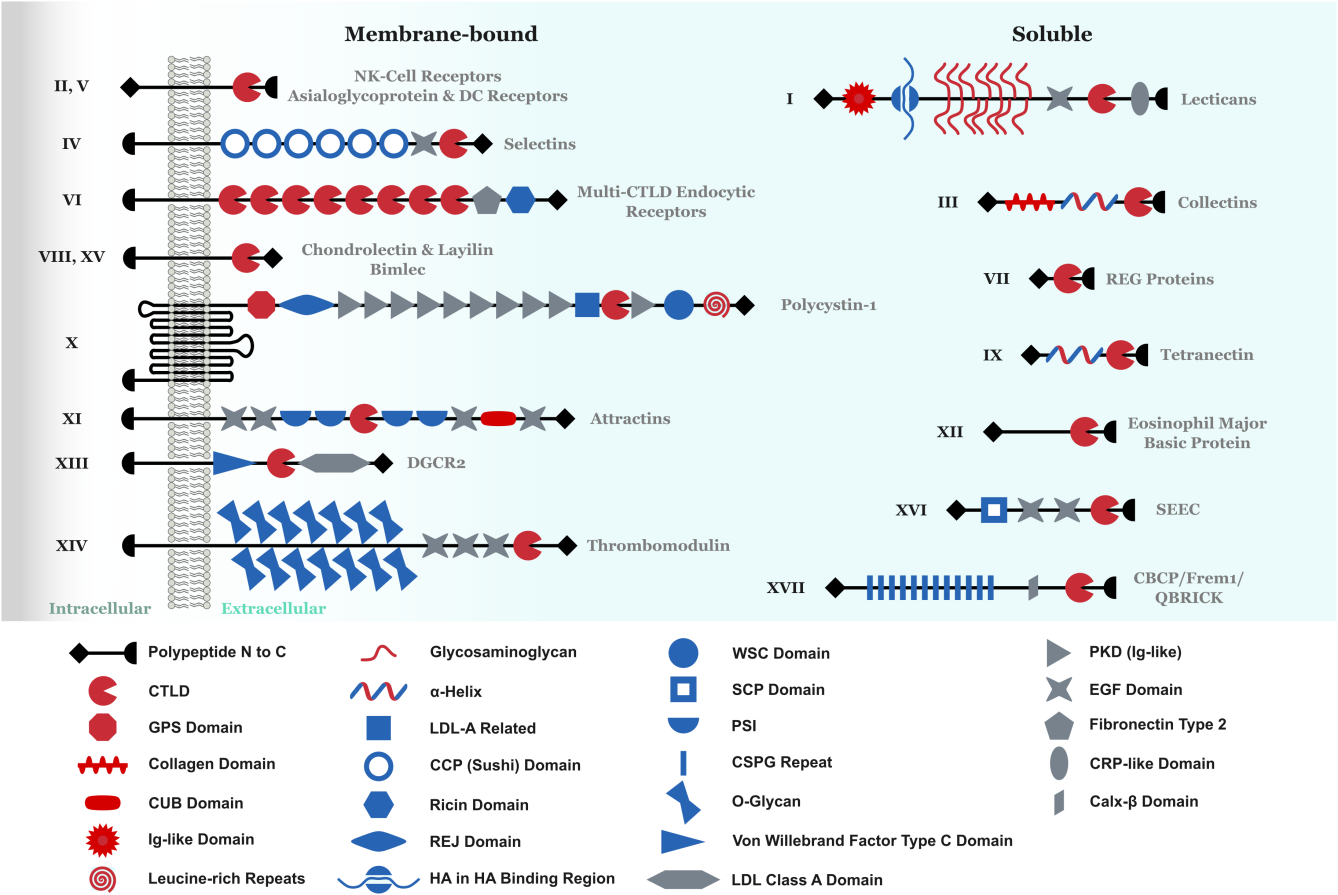
Schematic representation of the 17 groups of the C‐type lectin‐like domain superfamily with the distinction between soluble and membrane‐bound CLRs. Groups II and V contain many myeloid CLRs, e.g., DC‐SIGN, langerin, and Mincle in group II, whilst Dectin‐1 and LOX‐1 occupy group V [1]. Group VI notably includes the mannose receptor (MR). Members of many of the other groups are known for mediating cell adhesion (including groups VIII and XV), especially the selectins (Group IV); E‐, P‐, and L‐selectin are involved in leukocyte migration. Among the soluble CLRs, particularly well known in the context of the immune system, are the collectins (group III) for their roles in innate immunity and complement activation.

C‐type lectin receptors (CLRs) can be defined as proteins possessing at least one C‐type lectin‐like domain (CTLD) and are part of the CTLD superfamily. Seventeen groups are used to classify this superfamily, primarily based on structure with certain phylogenetic classifications (Figure 1) [1, 2, 3, 4]. One subset of this superfamily includes the myeloid CLRs, which are found on cells of myeloid lineage, e.g., macrophages, dendritic cells (DCs), and neutrophils [5, 6]. They belong primarily to groups II, V, and VI (Figure 1) [7] and act as pattern recognition receptors (PRRs), part of the innate immune system’s ability to sense and subsequently respond to danger signals [8]. These danger signals include motifs decorating invading pathogens, known as pathogen‐associated molecular patterns (PAMPs) or self‐derived components, e.g., glycolipids exposed during cell damage, known as damage‐associated molecular patterns (DAMPs) [9]. Once activated, PRRs are responsible for a plethora of immune functions, including cytokine/chemokine production and phagocytosis [5, 10, 11, 12]. With an increasing wealth of knowledge regarding glycan binding motifs for CLRs, afforded by highly sensitive techniques like X‐ray crystallography [13], nuclear magnetic resonance (NMR) spectroscopy [14, 15], and surface plasmon resonance (SPR) [16], the number of reports of synthetic CLR ligands has also grown. Each technique has advantages and disadvantages: For example, X‐ray crystallography allows detailed inspection of the interactions that mediate binding. However, these are the interactions in the crystal, not in solution, and may not fully reflect the native binding event; furthermore, the co‐crystallization of proteins and ligands is not trivial [17]. In contrast, NMR does allow the binding to be investigated in solution (therefore, solubility is a central concern) and can enable the interacting atoms on either binding partner to be determined [18]. However, isotopically labeled proteins/ligands are often required. SPR is a much faster method that also requires smaller amounts of sample, but depends on the immobilization of one partner, and therefore, avidity effects must be considered when evaluating results [19]. For a more detailed comparison of methods that are used to detect CLR–ligand interactions, the reader is directed to a recent review [20].

Aside from understanding how CLRs modulate immune responses, the therapeutic use of synthetic ligands is of great interest, for instance, as vaccine adjuvants, which is not without its challenges [21]. Adjuvants may increase the efficacy of vaccines by the selective targeting of vaccine antigens to antigen‐presenting cells as well as their direct activation. Since carbohydrate–lectin interactions are notoriously weak, one manner by which binding can be promoted is avidity, which is commonly exploited in adjuvant design [22, 23, 24, 25, 26]. Natural ligands are also often multivalently displayed, e.g., N‐glycans on human immunodeficiency virus (HIV) binding to dendritic cell‐specific Intercellular adhesion molecule‐3‐grabbing non‐integrin (DC‐SIGN) [27]. However, with synthetic glycans, this multivalent display must be tailored and can play a large role in establishing effective synthetic ligands.

This review surveys the rich landscape of synthetic ligands for some of the most relevant myeloid CLRs: DC‐SIGN, macrophage inducible C‐type lectin (Mincle), dendritic cell‐associated C‐type lectin‐1 (Dectin‐1) and langerin, with a discussion of detailed examples and the broader structural features of high‐affinity ligands. The importance of these molecules will be contextualized with overviews of pathogen recognition, signaling, downstream effects, and therapeutic uses. Where applicable, current limitations of the field will be discussed, and finally, a perspective on the future of the field will be offered.

## 2 Ligand Recognition

### 2.1 C‐Type Lectin‐Like Domain

Myeloid CLRs consist of three domains: the intracellular/cytoplasmic domain (important for signaling), the transmembrane region, and the extracellular region (important for ligand binding) [28]. The CTLD is located in the extracellular region and varies significantly among the myeloid CLRs, enabling the broad spectrum of recognition required to mount a protective immune response. CTLDs are 110–140 residue‐long domains where ligand binding occurs [29]. Whilst canonical CTLDs are characterized by Ca^2+^‐dependent carbohydrate binding sites, there are many CTLDs that bind ligands in a Ca^2+^‐independent manner (e.g., Dectin‐1) [30] and/or bind non‐carbohydrate ligands (e.g., lectin‐like oxidized low‐density lipoprotein receptor‐1 (LOX‐1) and myeloid inhibitory C‐type lectin‐like receptor (MICL)) [31, 32, 33]. For canonical CTLDs, Ca^2+^ is coordinated by a vicinal diol of a carbohydrate ligand, often 3‐OH and 4‐OH, leading to a distorted octahedral geometry at the metal center [34]. Specificity for either mannose‐type or galactose‐type ligands can be determined by the presence of either a Glu‐Pro‐Asn (EPN) motif or a Gln‐Pro‐Asp (QPD) motif, respectively [1, 34]. Aside from the primary carbohydrate binding site, secondary sites/interactions often influence selectivity, e.g., the secondary site of DC‐SIGN that accommodates the galactose moiety of Lewis^X^ [35] or the site that accommodates the second glucose molecule of trehalose dimycolate (TDM) in Mincle [36]. This description of a single CTLD and the corresponding binding affinity is not the full picture as many CLRs are also capable of multimerization, e.g., homotetramer formation by DC‐SIGN [37] or heterodimer formation by Mincle and macrophage C‐type lectin (MCL), making avidity a practical consideration [38, 39].

### 2.2 Multivalency

The presentation of the ligand to the lectin has an enormous effect on binding, and multivalency is commonly employed to overcome weak monovalent carbohydrate–lectin interactions and generate high‐affinity binders. Several factors lead to the observed binding improvements when going from monovalent to multivalent ligands. The glycoside cluster effect can enable the engagement of multiple binding sites, and the accumulation of the weak individual interactions leads to a much stronger overall interaction [24]. The bridging of carbohydrate binding sites between receptors can also lead to higher apparent binding [40]. Additionally, the higher ligand density offered by multivalent displays can reduce off rates as when one ligand dissociates, another one is in close proximity to bind, termed statistical rebinding [41]. A related effect, internal diffusion (“bind and hop/slide”) describes how a lectin can temporarily avoid fully dissociating by moving along the epitopes presented on the multivalent display [42].

Aside from the affinity of the binding epitope for the lectin, binding is affected by many variables arising from multivalent display. These include ligand density, directionality, linker length, and flexibility. In efforts to control these variables, several scaffolds are commonly employed, e.g., dendrons, dendrimers, clusters, polymers, glycoproteins, liposomes, and nanoparticles. As multivalency is not the focus of this review, readers are directed to several comprehensive reviews to supplement this brief discussion [23, 24, 25, 43, 44, 45, 46].

## 3 Signaling and Downstream Effects

**FIGURE 2:**
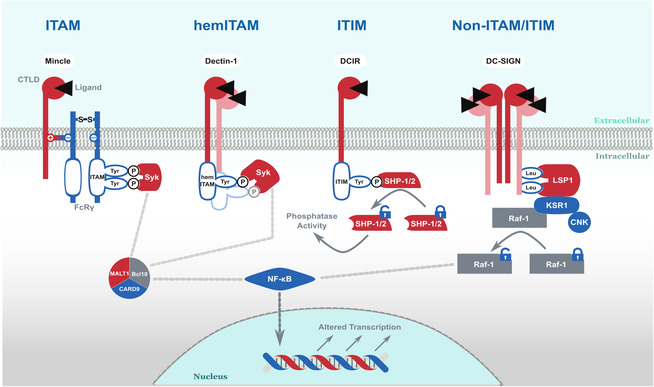
Summary of signaling from ITAM, hem‐ITAM, ITIM, and Raf‐1 pathways for representative CLRs. Mincle is shown to utilize the ITAM motif of an associated FcRγ chain to affect NF‐κB translocation via the Syk‐CARD9 axis. Dectin‐1 utilizes a hemITAM motif, which requires dimerization of the CLR to provide the phosphorylated binding site for Syk. The pathway utilized by DC‐SIGN (and also Dectin‐1) is distinct from the mechanisms of ITAM and hemITAM signaling as it relies upon the activation of Raf‐1. ITIM signaling requires just one phosphorylated tyrosine site (e.g., from the cytoplasmic tail of DCIR) to bind SHP‐1/2, activating it to engage in phosphatase activity. This counteracts other pathways that rely on phosphorylation to propagate signaling processes.

The binding of a ligand by a specific CLR can induce signaling, which is mediated by a variety of mechanisms. The most common include immunoreceptor tyrosine‐based activation motifs (ITAMs), hemi‐immunoreceptor tyrosine‐based activation motifs (hemITAMs), immunoreceptor tyrosine‐based inhibitory motifs (ITIMs), and the Raf‐1 pathway (Figure 2) [5]. FcRγ, a disulfide‐linked dimer possessing cytoplasmic ITAM motifs (Yxx(L/I)x_6–8_Yxx(L/I)), serves as an adaptor protein for many CLRs signaling via ITAMs [47]. The two tyrosine residues can be phosphorylated by Src family kinases (SRKs), a process enhanced by clustering [48]. Once these residues are phosphorylated, spleen tyrosine kinase (Syk) can bind and become activated. This leads to the formation of the ternary CARD9‐BcL10‐MALT1 complex that induces the activation and translocation of nuclear factor kappa B (NF‐κB) to the nucleus [49, 50]. In addition to NF‐κB activation, mitogen‐activated protein kinase (MAPK) pathways can also become activated [51, 52, 53].

HemITAMs are distinct from ITAMs, as they refer to single YxxL motifs in the cytoplasmic tail of certain CLRs, like Dectin‐1 or CLEC‐2 [54, 55]. As homodimers, each hemITAM can contribute one phosphorylated tyrosine to the binding site for Syk [55, 56]. Similar to ITAM‐mediated signaling, activation of the Syk‐Card9 axis leads to NF‐κB activation and MAPK signaling [57]. ITIMs can be represented generally by a I/V/LxYxxL/V motif [58]. For instance, DCIR contains a single ITIM motif in its cytoplasmic tail [59]. Phosphorylation of the tyrosine by SRK allows src homology 2 (SH2) domain‐containing inositol 5‐phosphatase‐1 (SHIP) or SH2 domain‐containing phosphatase‐1 or ‐2 (SHP‐1 or SHP‐2) to bind, which results in their activation [58]. Once activated, these phosphatases work to counteract activating signals, thus dampening inflammatory responses [58, 59]. Other CLRs, including DC‐SIGN, do not use ITAM or ITIM motifs; instead, they rely on the Raf‐1 pathway [60] (also used by Dectin‐1 [61]).

Ligation of a specific CLR by pathogen‐ or self‐derived ligands translates into a variety of cellular responses, including endocytosis, cytokine production, antigen presentation, and subsequent T cell activation [5]. While some CLRs are crucial for the induction of a proinflammatory response contributing to host protection, others may dampen cell activation to prevent a dysregulated immune response, otherwise leading to immune pathology. Hence, it is not surprising that CLR activation is regulated by crosstalk mechanisms with other PRRs in the innate immune system. The complexity of CLR responses to different ligands is also highlighted by the fact that CLR ligation may either lead to activation or inhibition of cellular responses in a context‐dependent manner.

The variability of CLR responses to different ligands and the often incompletely understood signaling pathways responsible for either myeloid cell activation or inhibition render CLR targeting a difficult task [5]. However, CLRs also have many favourable characteristics due to their distinct ligand preference [20], their defined expression pattern on myeloid cells, tailored markedly by the tissue microenvironment as well as specific PAMPs and DAMPs [5], and their importance in shaping and/or fine‐tuning of immune responses [62]. Hence, CLRs represent promising targets to enhance or modulate immune responses during infections and inflammatory processes. Signaling and downstream effects are exemplified below for the CLRs in the scope of this review.

### 3.1 DC‐SIGN

DC‐SIGN (also known as CD209) is classified as a type II transmembrane protein. It is primarily expressed by professional antigen‐presenting cells, particularly DCs, and certain macrophage populations [63]. Structurally, DC‐SIGN forms tetramers at the plasma membrane, with each subunit carrying a carbohydrate recognition domain (CRD) that enables interaction with distinct glycan motifs, including mannose‐ and fucose‐rich carbohydrates such as Lewis antigens. The CRDs are linked to a neck region composed of several highly conserved amino acid repeat sequences. This elongated neck structure promotes the tetramer formation at the cell surface [64]. Human DC‐SIGN has several orthologs in mice, including mDC‐SIGN as well as mSIGNR1 to mSIGNR4 [65]. DC‐SIGN contributes to a wide range of immune functions, including intercellular communication, antigen uptake and presentation via major histocompatibility complex (MHC) class I and II pathways, and modulation of intracellular signaling pathways [25]. The signaling outcome downstream of DC‐SIGN is highly dependent on the nature of the ligand encountered. DC‐SIGN signaling triggered by mannose‐containing ligands is mediated through the adaptor protein lymphocyte‐specific protein 1 (LSP1). Two leucine residues on the cytoplasmic tail of DC‐SIGN function as a site for LSP1 to bind, which then recruits kinase suppressor of Ras 1 (KSR1) and connector enhancer of KSR1 (CNK), which form a triad with Raf‐1 [63]. The activated Raf‐1 can activate the ERK MAPK pathway as well as NF‐κB [60, 66]. While activation of DC‐SIGN by mannosylated glycans results in NF‐κB translocation to the nucleus, thereby inducing proinflammatory gene expression, the binding of fucosylated glycans drives a distinct signaling program that suppresses immune activation and proceeds independently of Raf‐1. These contrasting effects demonstrate that DC‐SIGN can either enhance or dampen immune responses in a context‐dependent manner, thus underscoring the importance of the glycan microenvironment in regulating immune cell activation through DC‐SIGN [64].

### 3.2 Mincle

Mincle belongs to the family of type II transmembrane CLRs and is primarily present on innate immune cells of myeloid origin, such as macrophages, DCs, monocytes, and neutrophils [5]. Expression has additionally been reported in selected B‐cell subsets. As Mincle lacks intrinsic signaling capability, it associates with the FcRγ chain, leading to the recruitment of the tyrosine kinase Syk, followed by activation of the ternary CARD9–BCL10–MALT1 signaling complex, as described previously. This typically leads to the release of proinflammatory cytokines and chemokines such as tumor necrosis factor (TNF), macrophage inflammatory protein 2 (MIP‐2), and IL‐6 [62]. Cross‐talk between Mincle and Toll‐like receptors 7/8 in DCs further amplifies inflammatory signaling by promoting NF‐κB activity and inflammasome assembly, which enhances IL‐1β production [67]. Mincle activation drives Th17‐directed immune responses through the elevated expression of IL‐6, IL‐23, and pro‐IL‐1β [68]. Concurrent activation of the NLRP3 inflammasome enables proteolytic conversion of pro‐IL‐1β into biologically active IL‐1β. The expression of Mincle by antigen‐presenting cell subsets, e.g., macrophages, is context‐dependent and is markedly upregulated by certain PAMPs and proinflammatory cytokines, such as bacterial lipopolysaccharide (LPS) [69] and TNF [70], whereas it is inhibited by the Th2 cytokine IL‐4 [71]. Under certain conditions, Mincle engagement induces an inhibitory ITAM (ITAMi) configuration, thus impairing DC activation [72]. This immune regulatory role of Mincle can be exploited by pathogens for immune evasion, as shown for the *Leishmania* parasite that impairs cross‐presentation of parasite antigens to CD8^+^ T cells via Mincle‐ and SHP‐1‐dependent mechanisms [72, 73]. On the other hand, Mincle‐mediated regulation of innate responses can also be beneficial to the host and contribute to the maintenance of immune homeostasis through the production of the anti‐inflammatory cytokines IL‐10 and TGF‐β [74]. A recent study indicates that microbiota recognition by Mincle is also involved in a process called trained innate immunity, thereby leading to epigenetic and metabolic reprogramming of monocytes, macrophages, and bone marrow progenitors [75]. Therefore, Mincle ligation may not only lead to short‐term effects, such as immediate myeloid cell activation, but may also have a long‐lasting impact on immunity. A structurally related receptor, MCL, shares functional features with Mincle. This receptor is expressed by myeloid cell subsets and forms heterodimeric complexes with Mincle at the plasma membrane. Through this interaction, MCL may contribute to stabilization and surface retention of Mincle, thereby influencing its functional activity [76].

### 3.3 Dectin‐1

Classified within group V of the C‐type lectin superfamily, Dectin‐1 is a type II transmembrane receptor composed of an extracellular carbohydrate‐recognition domain, a stalk domain, a transmembrane region, and an intracellular tail containing signaling elements such as a hemITAM motif and a tri‐acidic sequence. Expression of Dectin‐1 is detected on several immune cell populations, including DCs, macrophages, neutrophils, and lymphocyte subsets [68]. A central consequence of Dectin‐1 ligation is activation of NF‐κB, which can proceed through a canonical and non‐canonical route. Ligand binding induces phosphorylation of the tyrosine residue within the hemITAM motif located in the cytoplasmic tail, thus enabling recruitment of Syk. Canonical Dectin‐1 signaling resembles classical ITAM‐mediated pathways and involves activation of PKCδ together with the CARD9–BCL10–MALT1 signaling complex. This Syk‐dependent NF‐κB activation drives a proinflammatory program in myeloid cells, characterized by DC maturation [77]. In parallel, Dectin‐1 can also signal independently of Syk via a pathway involving the Ras‐activated serine/threonine kinase Raf‐1, which likewise converges on NF‐κB activation [78]. Dectin‐1 signaling provokes numerous downstream cellular responses, including the release of proinflammatory cytokines, such as TNF [79]. Moreover, signaling via Dectin‐1 plays a key role in shaping adaptive immune responses by promoting the polarization of naïve CD4^+^ T cells into Th1 and Th17 effector cells, for instance, via IL‐6 and IL‐23 production [61]. Additional effector functions triggered by Dectin‐1 ligation include phagosome maturation, respiratory burst, neutrophil extracellular trap (NET) formation, DC maturation, and antigen presentation [80]. Hence, Dectin‐1 engagement not only affects initiated innate responses but also promotes adaptive immunity, in particular Th1 and Th17 responses [81]. For more details on Dectin‐1 signaling, the reader is referred to recent reviews [77, 82]. However, Dectin‐1 can also contribute to immune modulation, for example, in the context of cancer [83]. While immune suppression by Dectin‐1 during cancer is detrimental to the host, a modulated immune response may be beneficial to protect from autoimmunity and to maintain immune homeostasis [84]. Hence, Dectin‐1‐triggered responses may be context‐dependent and range from proinflammatory signaling to immune modulation.

### 3.4 Langerin

Langerin is classified as a membrane‐associated receptor within the group II family of C‐type lectins. Its structure includes four major regions: a short intracellular N‐terminal tail, a membrane‐spanning segment, an extracellular neck domain, and a carbohydrate‐binding region located at the C‐terminus [85]. The neck domain contains α‐helical coiled‐coil structures that promote assembly of the receptor into a trimeric complex, which enhances its capacity to interact with carbohydrate‐containing ligands. Langerin is characteristically expressed by Langerhans cells (LCs), a specialized population of DCs found mainly in the epidermis of the skin and mucosal surfaces [86]. Species‐specific differences in langerin expression have been described [87]: In humans, langerin is primarily associated with CD1a‐positive antigen‐presenting cells, whereas in mice its expression is more commonly linked to CD8α‐positive DCs. One of the key functions of langerin is the uptake of external antigens, followed by their transport into Birbeck granules, organelles unique to Langerhans cells, followed by antigen processing and presentation to T cells. Although the cytoplasmic region of langerin contains a proline‐rich sequence that may contribute to intracellular signaling, the receptor itself lacks conventional activating or inhibitory signaling motifs. However, as langerin can interact with other proteins inside the cytoplasm, langerin may modulate or mobilize cellular mechanisms required for defense against pathogens [88].

## 4 Therapeutic Applications

### 4.1 DC‐SIGN

**FIGURE 3:**
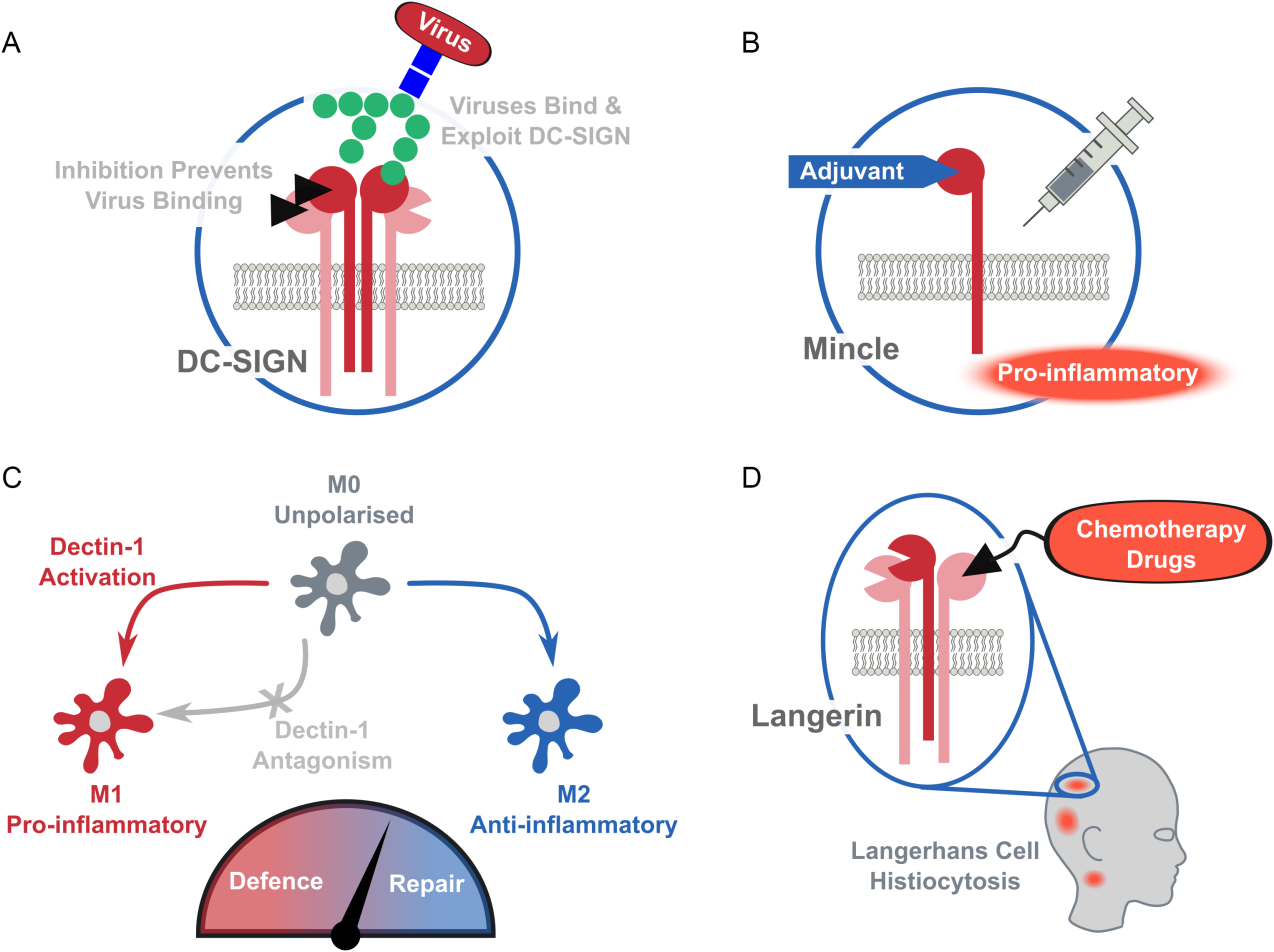
Summary of therapeutic applications targeting the selected myeloid CLRs. (A) The interaction between viruses and DC‐SIGN can be blocked to inhibit viral entry into host cells. (B) Mincle can be used as a target for adjuvants to improve the efficacy of vaccines. (C) Dectin‐1 can be activated or antagonized to modulate the immune response and favor either a more proinflammatory or anti‐inflammatory environment by influencing macrophage polarization. (D) The use of langerin targeting to deliver chemotherapy drugs to langerin‐expressing cells found in Langerhans cell histiocytosis.

The important role of DC‐SIGN in endocytosis, viral entry into host cells, and immune modulation render it a promising target for therapeutic applications. Indeed, numerous different applications have been reported, among the most widespread are decoy ligands to inhibit virus binding to DC‐SIGN. For example, the DC‐SIGN‐mediated *trans*‐infection by SARS‐CoV‐2 could be potently inhibited by a multivalent display of DC‐SIGN‐binding mannosides (IC_50_ = 1.46 nM) (Figure 3A) [89]. Despite numerous investigations and successes, translation from research into the clinic is not straightforward. One of the limiting factors is the development of high‐affinity binders that also display high selectivity. An illustrative example is the role of DC‐SIGN in promoting viral infection by HIV and Ebola, making it a promising target to inhibit [90, 91, 92, 93, 94]. However, the design of inhibitors is complicated by the fact that langerin has a similar binding specificity, yet langerin is protective during HIV infection [86]. In addition to its similarity to langerin, DC‐SIGN also shares 82% sequence identity with liver/lymph node‐specific ICAM‐3 grabbing non‐integrin (L‐SIGN), which has led to campaigns to establish selective ligands [95, 96]. This highlights the balancing act of CLR targeting owing to similarities in certain CLR binding specificities and their potentially antagonistic functions. Furthermore, interspecies differences between CLRs (preclinical animal species vs. humans) lead to difficulties in developing CLR‐targeting drugs. The human DC‐SIGN (hDC‐SIGN) and its murine orthologue SIGNR5 (mDC‐SIGN) share many functional aspects [97]; however, there are differences in ligand specificities [98]. Therefore, alternative systems to study DC‐SIGN in mice, e.g., humanized mouse models or hDC‐SIGN transgenic mice, can be beneficial [99].

Another well‐researched aspect of DC‐SIGN targeting is the cell‐specific delivery of vaccine antigens. For a vaccine to be successful, the antigenic motifs must be presented on MHC class‐I (to enable CD8^+^ T cell activation) and MHC class‐II molecules (to enable CD4^+^ T cell activation). This first involves antigen‐presenting cells, in particular DCs, which internalize and process the antigen before presenting peptide fragments on MHC class‐II molecules. Additionally, cross‐presentation can enable internalized antigens to be presented on MHC class‐I molecules for CD8^+^ T cell activation, which is especially important for immune defence against intracellular pathogens, such as viruses [100, 101]. Therefore, a challenge is to increase the presentation of vaccine‐derived peptide fragments on MHC molecules; this is where DC‐SIGN can be exploited. By conjugating the vaccine antigen to a DC‐SIGN ligand [102, 103] or an antibody that targets DC‐SIGN [104, 105], the vaccine can be more efficiently internalized by DCs and therefore more abundantly presented on MHC molecules. This effect has also been demonstrated using liposomes loaded with an antigen, with DC‐SIGN ligands (Lewis antigens) on the surface, where an enhanced CD4^+^ and CD8^+^ T cell response was seen [106].

DC‐SIGN can also act in a regulatory/suppressive capacity. DC‐SIGN activation can lead to IL‐10 production, which induces regulatory T cells (Tregs) [107]. Through this mechanism, DC‐SIGN has been shown to induce immune tolerance, in cooperation with TLR4 signaling, following transplantation, by recognizing fucosylated ligands (e.g., Lewis^X^) [108]. Tolerance can also be induced by the interaction between human milk oligosaccharides and DC‐SIGN, which can lead to a suppression of LPS‐induced responses by DCs [109]. Hence, utilizing the DC‐SIGN to promote anti‐inflammatory responses may offer treatment options for immune‐related disorders [110].

### 4.2 Mincle

This review has already touched upon the notion of employing CLR ligands as adjuvants to enhance the efficacy of vaccines. The need for adjuvants is especially pertinent for subunit vaccines and synthetic vaccines, where the often limited immunogenicity can result in reduced efficacy of a vaccine candidate [111, 112]. Despite this, these types of vaccines typically have an improved safety profile and higher stability compared to live‐attenuated vaccines [112, 113, 114]. Adjuvants targeting PRRs can enhance antigen internalization into antigen‐presenting cells and subsequent presentation of antigen fragments on MHC molecules to T cells, foster the expression of co‐stimulatory molecules, and promote T cell activation and polarization [115, 116]. The desired T cell polarization depends on the pathogen; cellular (Th1‐mediated) responses are more pertinent for intracellular pathogens such as viruses, intracellular bacteria, and parasites; however, humoral (Th2‐mediated) responses are often crucial for extracellular pathogens such as helminths and extracellular bacteria [117]. On the other hand, for mucosal pathogens, such as fungi, a Th17 polarization is often beneficial, illustrating the importance of selecting a suitable adjuvant for a given vaccine formulation [118]. Alum, an adjuvant consisting of aluminum salts, has been shown to induce strong Th2 polarization [117], whereas QS‐21 has been shown to induce Th1 polarization [119]. In practice, it is not purely a case of one or the other, but rather encouraging a mixed, robust immune response [120, 121]. Exploiting myeloid CLRs, especially Mincle, to affect adjuvant activity has been the subject of many campaigns that have been comprehensively reviewed (Figure 3B) [21, 122]. Among the most widely used is CAF01: a liposome adjuvant consisting of a surfactant and trehalose dibehenate (TDB) that targets Mincle, leading to a Th1/Th17 response [123, 124, 125, 126]. It has been tested in multiple vaccine formulations in clinical trials, including those against tuberculosis [127], malaria [128], chlamydia [129] and HIV [130]. Promisingly, similar adjuvant compositions with different Mincle ligands have been tested in mice and demonstrated the potential to induce stronger Th1 and Th17 responses than the TDB formulation [131].

### 4.3 Dectin‐1

Dectin‐1 has the potential to be leveraged therapeutically against various pathologies either agonistically (e.g., with curdlan or zymosan) or antagonistically (with laminarin), depending on the specific case (Figure 3C). One of the major therapeutic aspects of inhibiting Dectin‐1 is the ability to reduce inflammation. The activation of Dectin‐1 has been shown to polarize macrophages to a proinflammatory phenotype (M1) [132]. Therefore, the inhibition of Dectin‐1 represents a potential therapeutic intervention to reduce this and increase the proportion of anti‐inflammatory macrophages (M2), which aid in tissue repair. This has been demonstrated in injury models, where following injury, the expression of Dectin‐1 on macrophages increases and leads to a polarization of macrophages towards the proinflammatory phenotype and production of proinflammatory cytokines (e.g., TNF). However, when Dectin‐1 is inhibited, the polarization to the M1 phenotype is reduced, and clinical outcomes can improve. This has, for instance, been shown for intracerebral hemorrhage and ischemic stroke, where Dectin‐1 is expressed on microglia/macrophages [133, 134]. Following inhibition of Dectin‐1, neurological dysfunction was reduced. Furthermore, cardiac function was improved by inhibiting Dectin‐1 following myocardial ischemia/reperfusion injury, which is a complication of primary percutaneous coronary intervention [135].

In terms of therapeutic activation of Dectin‐1, treatment with an agonist was shown to promote central nervous system axon regeneration following retro‐orbital optic nerve crush [136]. This effect was even seen after a 2‐day delay in administering the agonist, which could represent a favorable therapeutic window. However, toxicity was also noted, possibly linked to the interaction of Dectin‐1 with its ligand curdlan. In another study, Dectin‐1 agonism by *Ganoderma lucidum* polysaccharides was observed to have an antidepressant effect in mice that was inhibited by the antagonist laminarin [137].

### 4.4 Langerin

Owing to the largely restricted expression of langerin to Langerhans cells (LCs), the therapeutic opportunities of langerin targeting center on the delivery of compounds to LCs, e.g., vaccines. Anti‐langerin monoclonal antibodies fused with HIV antigens have been developed as potential HIV vaccines and were found to promote a humoral response, including increasing T follicular helper cell (Tfh) numbers, B cell activation and IgG production [138]. One impressive aspect of LC targeting via langerin is the possibility to deliver antigens to LCs through microneedle injection, owing to the presence of LCs in the epidermis and dermis [139]. In addition to vaccines, the delivery of drugs to LCs can be necessary, e.g., to kill abnormal LCs (Figure 3D). Liposomes loaded with the cytotoxic drug doxorubicin and decorated with glycomimetic langerin ligands were found to selectively target LCs and induce doxorubicin‐mediated killing [140]. This represents a potential avenue for the treatment of Langerhans cell histiocytosis, a cancer(‐like) condition, causing lesions that can be local or disseminated, which would benefit from a targeted treatment [141].

## 5 Synthetic Ligands

A number of synthetic ligands have been designed and validated, which are mainly orthosteric ligands, based on knowledge of the core binding requirements of a given CLR with derivatization of the peripheral groups [142]. Allosteric ligands have also been identified for certain CLRs and can have the benefit of targeting lectins with hydrophilic primary sites using more traditional drug‐like molecules [143].

The binding of ligands to lectins is often evaluated by the K_D_ (equilibrium dissociation constant) or IC_50_ (ligand concentration to inhibit 50% of activity) value derived from a direct binding determination (e.g., SPR or NMR spectroscopy) [23], but may also be demonstrated by cell‐based assays. Natural ligands typically have weak interactions with lectins, often in the millimolar (for monosaccharides) or micromolar (for oligosaccharides) range [144]. However, in the search for potential therapeutics, these affinities are usually not sufficient. Millimolar to moderate micromolar affinity is relatively weak binding but can indicate promising fragments, whereas low micromolar affinity is considered a promising initial lead [145]. Generally, low nanomolar affinities and the sub‐nanomolar range are considered a well‐optimized lead compound [146]. Therefore, the goal of many reports is to develop high‐affinity monovalent binders, which can then be further enhanced by avidity through a multivalent display to achieve drug‐like binding affinities.

### 5.1 DC‐SIGN

#### 5.1.1 Structure of DC‐SIGN

DC‐SIGN possesses a canonical site and binds glycans in a Ca^2+^‐dependent manner. The EPN motif means that these are commonly mannose‐ and fucose‐type ligands. For high mannose‐based structures, it is the equatorial/equatorial 3‐OH/4‐OH diol that coordinates the Ca^2+^ whereas for fucose‐based ligands, it is the equatorial/axial 3‐OH/4‐OH [35]. Aside from interactions with the primary site, natural and synthetic ligands target a number of secondary sites to stabilize their binding. The isopropyl group of Val351 can engage in van der Waals interactions, whereas the primary alcohol of Ser360 can hydrogen bond and the phenyl ring of Phe313 can engage in π‐interactions [147, 148]. In addition to the CRD, the extracellular region of DC‐SIGN incorporates a neck domain. Interactions between the neck domains of different DC‐SIGN molecules enable the equilibrium formation of tetramers, and the consequent avidity enhances the strength of ligand binding [37].

**FIGURE 4:**
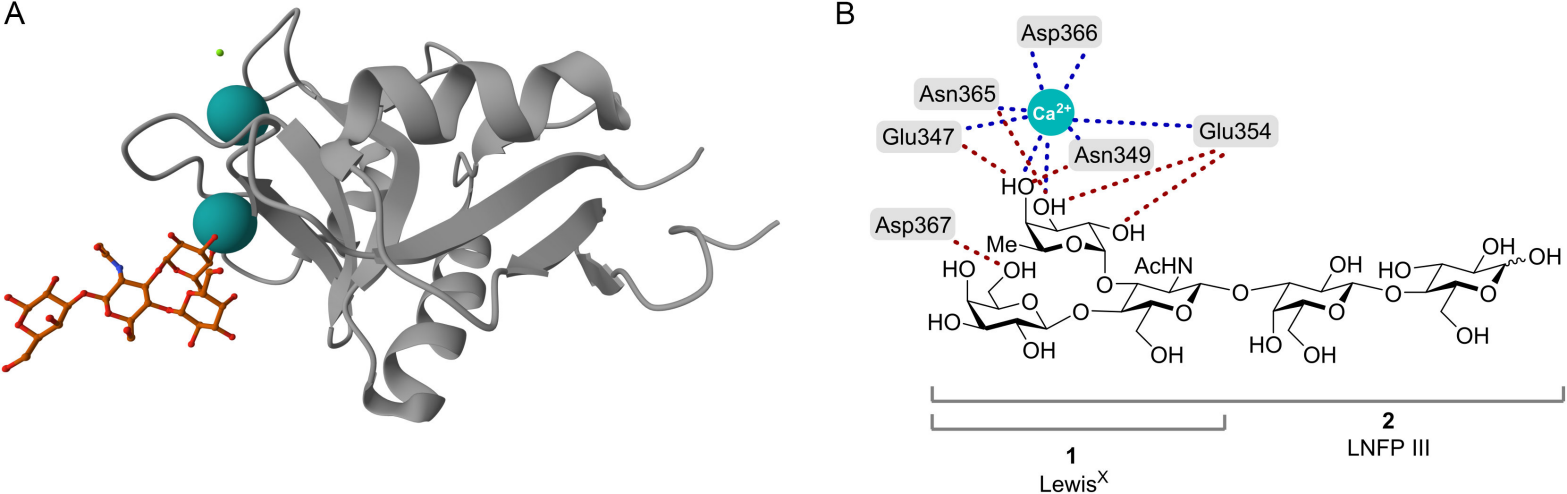
(A) Crystal structure of human DC‐SIGN (gray) binding LNFP III (orange/red) (PDB ID: 1SL5) [35], visualized with Mol*viewer [149]. Ca^2+^ ions (blue) have been enlarged for clarity. (B) Ligands of DC‐SIGN: LNFP III and its substructure Lewis^X^. Cartoon showing interactions with DC‐SIGN, where blue lines indicate interactions with calcium and red indicate other key protein–ligand interactions.

For fucose‐containing ligands such as Lewis^X^ **1** and LNFP III **2** (PDB: 1SL5, Figure 4), the binding is further stabilized by hydrogen‐bonding networks (e.g., with Glu347, Asn349, Glu354, and Asn365) and van der Waals interactions (e.g., with Val351) [35]. The terminal galactose residue occupies a secondary site to further stabilize the binding through direct (C6‐OH and Asp367) and water‐mediated hydrogen bonding and van der Waals interactions (C6‐OH and Leu371).

DC‐SIGN is one of the most researched CLRs, and therefore, there exists a wealth of literature aimed at designing high‐affinity binders. The majority of synthetic ligands are based on two classes of natural ligands: high mannose structures (e.g., those found on the gp120 glycoprotein of HIV) [27] and fucose‐containing Lewis antigens (such as Lewis^X^ **1**) (Figure 4) [150]. The common workflow involves identifying a high‐affinity monovalent ligand and then enhancing the binding through avidity gains with multivalent presentations, including dendrons, dendrimers, gold nanoparticles, oligolysine clusters, and others [151, 152, 153, 154, 155, 156, 157, 158, 159]. In addition to the improved avidity, these multivalent constructs can facilitate further functionalization, e.g., dendrons also carrying a BODIPY dye [160]. Though many of the ligands fall into three major categories (carbohydrates/glycomimetics, allosteric ligands, or covalent ligands), there are exceptions. Malonates have been observed to bind DC‐SIGN by coordinating Ca^2+^ [161]. Difluorinated malonate **3** (*K*_D_ = 1.2 mM) was found to interact with the primary Ca^2+^ site, seen by a chemical shift perturbation (CSP). A CSP of Phe313 was attributed to the electronegative CF_2_ group, an interaction that was predicted during the design of the compound.

#### 5.1.2 Mannose‐Based DC‐SIGN Ligands

As is common in synthetic ligand design, the smallest functional molecule is sought, which in this case could be thought of as d‐mannose itself. Despite the relatively unimpressive binding profile of d‐mannose (*K*_D_  =  3.5 mM), it could be manipulated into a promising lead, e.g., by multivalent quantum dot presentation (*K*_D_  =  0.6 nM) [162].

**FIGURE 5:**
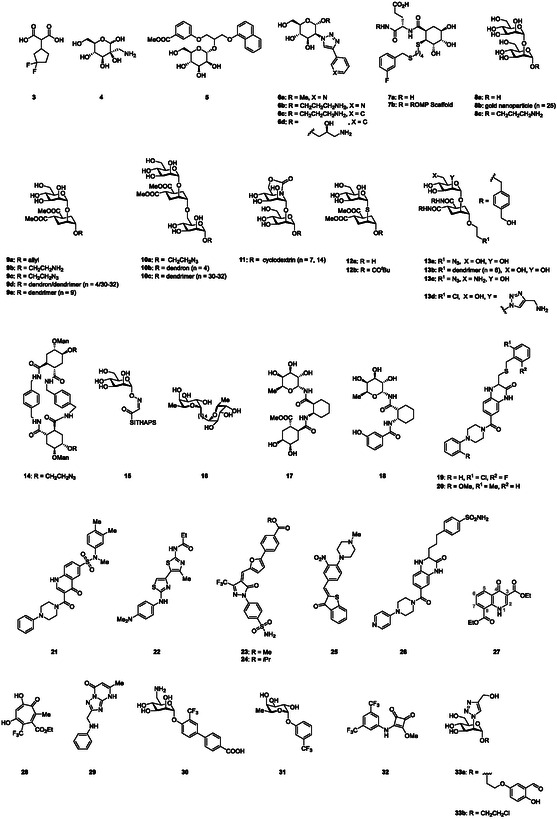
Structures of selected and relevant synthetic DC‐SIGN ligands. A variety of core structural features are seen that include malonate **3**, mono‐ (**4**‐**7**), di‐ (**8**,**9**,**11**‐**13**), and trisaccharide (**10**) mannose mimics as well as fucose mimics **16**‐**18**. Allosteric (**19**‐**31**) and covalent (**32**‐**33**) ligands are also shown.

Nevertheless, efforts to find high‐affinity mannose mimics led to reports of structures **4**‐**7** (Figure 5). Aminomethyl compound **4** exhibited a 48‐fold improvement in binding compared to d‐mannose [163]. Modifications to the anomeric substituent of mannose have also been investigated, e.g., aryl substituted glycerol [164], allowing **5** (*K*_D_ = 0.45 mM) to be identified as a ligand [165]. The choice to include the aryl moieties was deliberate to target the hydrophobic secondary site around Phe313, and the glycerol linker was chosen, as in docking studies, it offered a suitable length for the aromatic portion to reach Phe313. Also to target an interaction with Phe313, docking of **6a** predicted a binding orientation where the 6‐membered aromatic ring would contact Phe313 [148]. However, the crystal structure (PDB: 7NL6) revealed that the binding was rotated, instead allowing this contact to be made to Val351. Modifications led to **6b** (*K*_D_  =  29 μM) and **6c** (*K*_D_  =  32 μM), both demonstrating more than a 100‐fold improvement over methyl α‐d‐mannopyranoside. The crystal structure of **6c** (PDB: 7NL7) showed that the aromatic group again interacted with Val351 and that the anomeric aminopropyl linker enabled hydrogen bonding with Ser360 and Glu358. Attempts to conformationally restrict the amine (cyclization) succeeded in reducing the entropy penalty of binding, but resulted in impaired ligand–protein interactions and corresponding enthalpic penalties, resulting in no improvement of binding affinity [147]. Attention instead focused on optimizing hydrogen bonding with Ser360 and Glu358, leading to the (*S*)‐configured aglycone of **6d** (*K*_D_  =  9 μM). The stereochemistry of such mimics is not always set by using carbohydrate starting materials; other chiral building blocks have been successfully employed. Shikimic acid‐based glycomimetics that target the CRD have also been investigated, and it was shown that a multivalent display of these mimics also improved binding [166, 167]. The inhibitory ability of **7a** could be increased ∼1000‐fold by multivalent display on a ring‐opening metathesis polymerization (ROMP)‐derived scaffold **7b** (IC_50_  =  2.9 μM). Whilst the majority of monosaccharide structures discussed here could impressively achieve low micromolar affinities, significant attention has been devoted to disaccharide motifs in efforts to obtain even stronger binding.

Many synthetic mannose‐based DC‐SIGN ligands are derivatives of the disaccharide Manα1‐2Manα **8a**. Complete inhibition of DC‐SIGN was seen at just 115 nM with nanoparticle **8b** (vs. 2.2 mM for monomeric **8c**) [168]. The limited stability of glycans under physiological conditions is one of the major limitations of glycan‐based therapeutics, but more stable analogs have the power to improve the practical applicability of a glycan [169]. To this end, the reducing‐end mannose motif of **8a** was modified by exchanging the pyranose ring for a substituted cyclohexane ring. This effectively removed the glycosidic nature of the linkage to the alkyl amino/azido linker [170]. The ring substituents were also modified to include only the glycosidic linkage to the nonreducing end mannose and a bis‐methyl ester motif. Gratifyingly, **9a** proved to be ∼6‐fold more stable to mannosidase hydrolysis compared to **8a.** Furthermore, **9b** demonstrated a ∼3‐fold higher inhibition vs. the natural dimannoside in an infection model [171]. The related trisaccharide **10a** (IC_50_  =  125 μM) was found to have a significantly higher affinity for DC‐SIGN than **9c** (IC_50_ = 1.0 mM) by SPR measurement, and tetrameric dendron **10b** led to a 16‐fold improvement (vs. monovalent **10a**) in inhibition during an infection study [172]. The crystal structures of **9b** (PDB: 2XR5) [92] and **10a** (PDB: 2XR6) [173] were very similar, revealing the 2‐OH of the nonreducing end mannose interacting with Asn365 and a water‐mediated hydrogen bond network to Asp367. As for other mannose ligands, as well as coordinating the Ca^2+^, 3‐OH and 4‐OH interacted with Glu354/Asn365 and Asn349/Glu347, respectively. A van der Waals interaction between Val351 and the C6 methylene of the cyclohexane ring was also observed. Despite the SPR results indicating monovalent **10a** to be a significantly higher‐affinity binder than **9c**, the multivalently displayed analogs **9d** and **10b/10c** performed similarly in infection studies. It was then determined that monovalent **10a** was able to engage two separate DC‐SIGN CTLDs, which **9c** could not, yet the possibility of this binding mode was abolished upon conjugation to the dendron/dendrimer. This illustrates the power of altering presentation modes and how it can influence binding modes. Higher‐valent displays also offered stronger binding, with nonamer **9e** having a ∼60‐fold higher potency than the monovalent ligand **9b** [174].

The (*pseudo*‐)glycosidic linkage (a metabolically labile bond) can be targeted to modulate metabolic stability of lectin ligands, and iminosugars have been shown to be less susceptible to chemical and biological cleavage [175]. Multivalent displays have been employed with sp^2^‐iminosugar mimetics of Manα1‐2Manα presented on (hepta‐ or tetradecavalent) β‐cyclodextrin rings as DC‐SIGN ligands with improved stability [176]. The best‐performing mimetic motif in this study (determined by SPR) contained the iminosugar at the non‐reducing end, with mannose at the reducing end (**11**). Additionally, editing positions outside the ring can also lead to gains in metabolic stability, e.g., S‐linkages [177]. This modification was shown to be tolerated in terms of DC‐SIGN binding; S‐linked **12a** and **12b** were approximately equipotent to **9c** [178].

Whilst amides can be more metabolically stable than esters [179], the choice to exchange the methyl ester groups of **9c** for amides was made with a view to maximize affinity by aiming to target the hydrophobic region near Phe313 with lipophilic moieties on the amides. **13a** (IC_50_  =  325 μM) was identified as particularly promising [93, 180]. The benefit of multivalency was also clear in this case; hexavalent dendrimer **13b** proved ∼50‐fold more potent than the monomer **13a** [174], and the use of alternative multivalent scaffolds (e.g., rod‐spaced dendrimers) has also been explored [181, 182]. Screening studies also suggested that tertiary amides were not well tolerated [183]. It was found that OH → NH_2_ substitution at the 6‐position of the non‐reducing‐end mannose, **13c** (IC_50_   =   254 μM), also improved binding compared to the natural Manα1‐2Manα (IC_50_   =   915 μM) but was relatively similar to the potency of **13a** (IC_50_   =   329 μM) that lacked the amine at C6 [90, 184]. An ammonium binding region was identified by virtual screening near Phe313 on DC‐SIGN and led to the targeted development of ligand **13d** that was more potent than **13a** and **13c** [185, 186]. The crystal structure of **13d** bound to DC‐SIGN (PDB: 6GHV) [185] showed the coordination of Ca^2+^ by the 3‐OH and 4‐OH of the mannose residue and the expected hydrogen‐bonding interactions for these two positions, and the van der Waals contact with Val351 was maintained. The ammonium group, as predicted, showed a cation‐π interaction with Phe313, as well as hydrogen bonds to Ser360 and Glu358. Furthermore, the amide carbonyl engaged in a hydrogen‐bonding interaction with Lys368. Despite dimer **14** having been identified as a strong binder (IC_50_  = 31 μM) during these studies, it was not pursued owing to synthetic challenges [93].

With the widespread use and high efficiency of solid‐phase peptide synthesis [187], ligand design based on the modification of the peptide unit of small glycopeptides is an attractive strategy. A genetically encoded library was used to screen for DC‐SIGN binding fragments and led to the development of a trivalent display of glycopeptide **15** (IC_50_   =   0.45 mM) that was also found to bind DC‐SIGN on the cell surface, enhanced by multivalent display [188].

#### 5.1.3 Fucose‐Based DC‐SIGN Ligands

Similarly to d‐mannose, l‐fucose is a poor DC‐SIGN ligand; however, by using multivalent displays of fucose (e.g., dendrimers, gold nanoparticles and calixarenes), binding was dramatically improved [189, 190, 191, 192]. Mimics of l‐fucose include C‐glycoside **16**, containing two fucose rings linked via a butyl linker. **16** was found to inhibit DC‐SIGN (IC_50_   =   0.43 mM by SPR), twice as potent as **1** [193]. Furthermore, the C‐glycoside would be expected to exhibit a higher metabolic stability, which was a driving force behind its design [194].

Fucose‐containing ligands of DC‐SIGN, like Lewis^X^ **1,** provide a sensible starting point from which novel ligands have been developed. Again, with enzymatic hydrolysis in mind, many mimics seek to offer improved stability. One example is the exchange of the glycosidic bonds of **1** for amides and replacing the reducing‐end GlcNAc with a cyclohexane‐based linker [195]. These changes required other alterations to maintain the overall 3D structure, and therefore, the galactose moiety also had to be exchanged for a mimic to avoid distorting interactions with the linker, yielding **17**. The inhibitory ability of **17** (IC_50_   =   0.35 mM) was superior to that of **1** (IC_50_   =   0.8 mM). The galactose mimic was observed to exert a less significant effect on binding, and therefore, this motif became the focus of subsequent derivatization [196] and **18** was found to be a promising ligand, with binding found to be sensitive to the stereochemistry of the cyclohexane linker [91].

#### 5.1.4 Allosteric DC‐SIGN Ligands

In addition to derivatizing known ligand classes or improving binding through multivalent displays, the search for entirely novel monovalent DC‐SIGN inhibitor classes as allosteric inhibitors has been fruitful [197, 198]. Screening compound libraries can offer a pipeline of potential inhibitors and lead to the identification of novel inhibitors **19**‐**25** (IC_50_   =   1.6 to 32 μM) [199]. The exact binding site was not determined at this stage. Substructures of **20** were found to bind allosteric sites of DC‐SIGN, albeit with lower affinity [197, 200]. The oxidizable thioether of quinoxalinone **20** was suspected to be a stability weak point and was targeted for derivatization [201]. A panel of methylene derivatives led to the identification of **26** as a stable alternative with good affinity (IC_50_   =   0.31 μM). Key aspects of the design included an aromatic group on the piperazine ring, especially a nitrogen heterocycle. Furthermore, binding was diminished when the quinoxalinone nitrogen was substituted. An aromatic group on the propyl linker was beneficial and the effect was maximized when it bore a polar group, e.g., SO_2_NH_2_. Structurally related quinolones have also been identified to bind DC‐SIGN using NMR [202]. In this case, binding is reported to be allosteric in nature, though no specific site was identified, and several trends were observed during the SAR study. DC‐SIGN binding was promoted by a dimethylamino carbonyl group or an ethoxycarbonyl group at position 3, and the effect was even stronger with accompanying substituents at position 7/8. Many of the compounds in this study suffered from poor solubility and therefore limited the number of structures that could be tested for binding. Despite this, several moderate‐affinity binders were identified, the strongest being **27** (*K*_D_   =   0.1 mM). The power of NMR screening is even greater when an NMR‐active nucleus with high sensitivity and a broad chemical shift range is used, like ^19^F‐NMR, as this can allow multiple fluorinated ligands to be screened simultaneously [203]. This strategy was employed to screen a library of 95 fragments, portioned into four groups of 23/24 fragments [204]. By screening these portions with DC‐SIGN, hit compounds were identified with ^19^F‐NMR. Cycloheptatriene **28** (*K*_D_   =   0.15 mM) was found to be the highest affinity binder, and the binding site was determined to be allosteric at Met270, Ser310, and Phe374.

Though fragment‐based screening with NMR offers a rapid initial method to identify potential ligands, these hits are then often validated by SPR and cell‐based assays, where a more realistic presentation of ligands and/or protein is possible [200]. In particular, cell‐based screening represents a more physiologically relevant environment and has been used to identify motifs bound by DC‐SIGN [205]. SAR studies can then be used to develop the identified hits into higher‐affinity binders, e.g., **29** (IC_50_   =   39 μM).

Furthermore, liposomes containing d‐mannose and **30** were determined to interact promiscuously with the canonical binding site or a secondary remote pocket near Met270 and Tyr268 [206]. This site was the subject of a later investigation and found to be a hydrophobic allosteric pocket that includes Thr261, Tyr268, Phe269, Met270, Phe302, Leu303, and Ile376 [207]. It was suggested that occupation of this allosteric pocket modulated binding in the extended carbohydrate binding region, e.g., at Phe313, but did not have any significant effects on the canonical carbohydrate site. A panel of aryl α‐l‐fucosides was examined via NMR and *meta*‐trifluoromethyl compound **31** (*K*_D_   =   600 μM) was most promising, showing good results in a plate‐based competition assay (IC_50_   =   730 μM) and an interaction with the allosteric site at Met270 [208].

#### 5.1.5 Covalent DC‐SIGN Ligands

In addition to ligands, the lectins themselves can also be modified, as their covalent modification can modulate lectin function. Compound **32** was indicated to specifically target Lys373 of DC‐SIGN, forming an amide bond, and altering the canonical binding site, which led to an increase in binding affinity for monosaccharides [209]. Reversible imine formation between lysine and salicaldehyde has been exploited to generate ligands with specificity for DC‐SIGN over L‐SIGN such as **33a** [210]. The choice to place the salicaldehyde motif in the C1 position was in order to target one of the two neighboring Lys residues established previously [185] (Lys368 over Lys373) because the ammonium of Lys368 was calculated to be the more acidic of the two. An ethylene glycol linker was employed to impart a degree of flexibility to the ligand so that imine formation would not disrupt the binding of the carbohydrate portion. A competition assay established that **33a** (IC_50_   =   93 μM) had an IC_50_ more than 7‐fold lower than **33b**, which lacked the salicaldehyde motif. However, in contrast to the computational predictions, mass spectrometry revealed it was actually Lys373 that interacted with the aldehyde of the salicaldehyde motif, which would be computationally possible when the carbohydrate is inverted in the primary site. Hence, from both of these studies, it appears that Lys373 is a validated target for the (reversible) covalent modification of DC‐SIGN.

Such approaches, assuming sufficient specificity for the target lectin, could possibly provide a method to deliberately skew immune responses in the context of adjuvanticity by fostering the engagement of certain lectins over others.

### 5.2 Mincle

#### 5.2.1 Structure of Mincle

**FIGURE 6:**
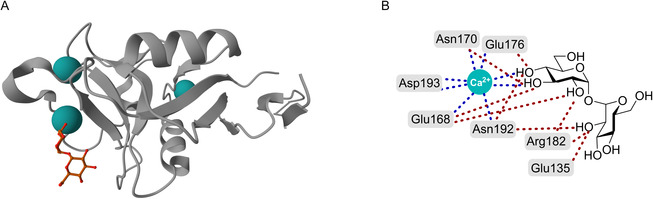
(A) Crystal structure of bMincle (gray) binding trehalose (orange/red) (PDB ID: 4ZRW) [36], visualized with Mol*viewer [149]. Ca^2+^ ions (blue) have been enlarged for clarity. (B) Schematic representation of the key binding interactions. Shown in blue is the coordination of the protein and ligand to the Ca^2+^ ion. In red are other key ligand–protein interactions.

The crystal structure of bovine Mincle (bMincle) (Figure 6) shows three Ca^2+^ sites; yet, only one of these is involved in binding. The remaining Ca^2+^ ions in Mincle serve to stabilize the conformation [36]. The calcium ion binding trehalose is situated in the “long loop,” which refers to a sequence of amino acids where ligand binding occurs [1]. bMincle binds carbohydrates where the 3‐OH and 4‐OH vicinal diol coordinate Ca^2+^ and neighboring residues (Glu168, Asn170, Glu176, Arg182, Asn192 and Asp193) stabilize the binding through hydrogen bonding to the hydroxyl groups of the carbohydrate, as seen for trehalose binding bMincle (PDB: 4ZRW, Figure 6) [36]. The second glucose moiety interacts with Asn192, Arg182, and Glu135 to stabilize the binding. Mincle also binds glycolipids, which is facilitated by the presence of a hydrophobic lipid binding groove adjacent to the primary carbohydrate binding site, which, for hMincle, includes Phe198, Leu199, Ile173 and Ala174 [211].

#### 5.2.2 Mincle Ligands with Linear Chains

**FIGURE 7:**
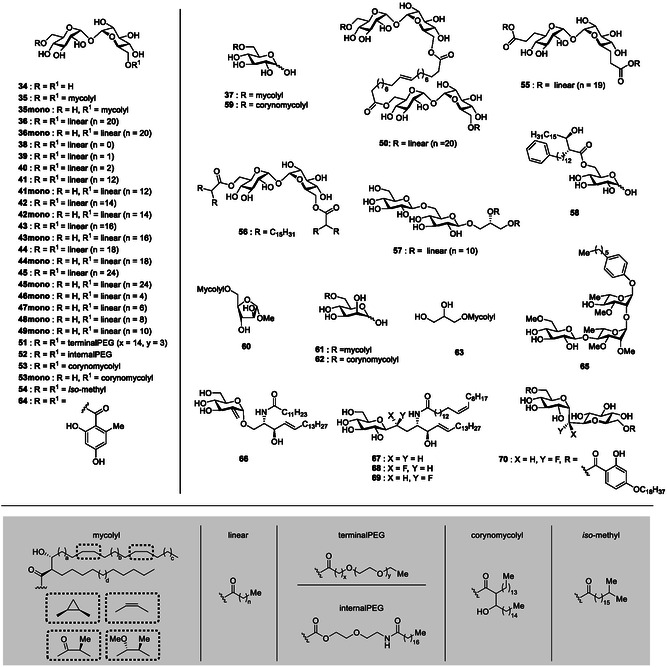
Structures of selected relevant synthetic Mincle ligands. Trehalose (**34**) has been derivatized extensively leading to a diverse panel of structures. Prototypical ligands TDM (**35**) and TDB (**36**) have been supplemented by libraries designed to investigate the effects of chain length, a selection of which are discussed here, including **36**‐**49**. Varied lengths of mono‐acylation have also been explored via the **mono** compounds, as well as hydrophilic chains (**51**, **52**), branching effects (**53**, **54**), and aromatic groups (**58**, **64**, **65**, **70**). Various compounds not containing α‐trehalose cores are also shown.

The most well‐known Mincle ligands are trehalose(**34**)‐based glycolipids, of which TDM **35** and TDB **36** (Figure 7) are most commonly encountered in the literature [212, 213]. In early reports of TDM and trehalose monomycolate (TMM) **35mono** activating Mincle, it was also suggested that mycobacteria transform TDM to glucose monomycolate (GlcMM) **37** to evade the host immune system [214, 215]. This was supported by the fact that trehalase‐treated TDM was a less potent activator of Mincle NFAT cells. However, doubt was cast on this theory as human Mincle (hMincle) and murine Mincle (mMincle) HEK reporter cells were more potently activated by GlcMM than TDM [131]. This was supported by another report that demonstrated GlcMM could activate bone marrow‐derived DCs (BMDCs), dependent on Mincle [216].

Compared to glucose, the trehalose motif offers an improved binding affinity; a 36‐fold increase is observed when going from α‐methyl glucoside to α‐trehalose **34** [217]. TDM **35** is a heterogenous glycolipid including two mycolic acids that can vary in length and also the presence of cyclopropane rings, vicinal carbonyl‐methoxy, and methoxy‐methyl motifs. Conversely, TDB **36** has a defined structure with a linear C_22_ lipid chain. The effect of chain length is one of the most well‐studied aspects of Mincle ligands, yet unfortunately, this has not translated to a definitive rule for ligand design as the effects vary significantly based on the core structure. Among the trehalose‐based linear diesters that have been investigated are the C2 (**38**), C3 (**39**), and C4 (**40**) analogs that showed longer chains moderately increased bMincle affinity, up to 8‐fold for **40** [217]. Larger structures have also been tested, including C_14_ (**41**), C_16_ (**42**), C_18_ (**43**), C_20_ (**44**), which bound mMincle similarly, with the exception of shorter **41** showing weaker binding [218].

In contrast to the diesters, the analogous monosters **41mono**, **42mono**, **43mono**, and **44mono** were found not to bind significantly to mMincle‐Fc proteins [218]. This activity/inactivity juxtaposition permeated to the compounds’ abilities to activate murine bone marrow‐derived macrophages (BMMs), with monoesters only affecting significant activation at high concentrations (∼100‐fold higher than diesters). However, the C_22_ and C_26_ diesters (**36**, **45**) and monoesters (**36mono**, **45mono**) showed a similar ability to activate murine BMMs [219]. Taken together, it appears that for shorter linear alkyl chains, two alkyl chains are required to activate Mincle. However, for longer chain lengths, the monoesters become comparable to the diesters. This is supported by the increase in binding to bMincle seen when increasing the chain length of trehalose‐based monoesters [217]. Inclusion of a C_6_ alkyl chain (**46mono**) afforded a 12‐fold increase vs. trehalose. Furthermore, a C_8_ alkyl chain (**47mono**) yielded a 52‐fold increase. Longer chain monoesters were also investigated with hMincle using SPR and it was found that C_10_ (**48mono**) and C_12_ (**49mono**) monoesters bound much more strongly than the C_8_ monoester (**47mono**) [220].

In an effort to produce a Mincle ligand that enhanced clustering by bridging two CRDs, **50** (an alkyl‐linked dimer of two TDB molecules) was designed [221]. The ability of **50** to signal through mMincle was confirmed with an NFAT reporter cell assay.

From a practical point of view, classic Mincle ligands can present certain problems, e.g., poor water solubility owing to the long lipid chains. To address this limitation, terminal polyethylene glycol (PEG) chains were trialed to improve water solubility [222]. In a binding study with mMincle‐Fc, **51** had a greater affinity than TDM **35**. Additionally, mMincle NFAT reporter cell assays showed signaling; however, no significant cytokine production in GM‐CSF‐differentiated bone marrow cells was observed. With an internal PEG unit, C_18_ diester **52** only bound mMincle‐Fc protein at high concentrations and did not lead to significant activation of BMMs or BDMCs [218]. Though a promising avenue, as of yet, the inclusion of PEG units requires further investigation.

#### 5.2.3 Mincle Ligands with Branched Chains

As well as linear chains, branched analogs of α‐trehalose glycolipids were also investigated; among these were the corynomycolates [223]. These corynomycolates were stated to be simpler to access synthetically compared to more complex heterogeneous mycolates. Trehalose dicorynomycolate **53** and monocorynomycolate **53mono** were both found to enact significant activation of hMincle and mMincle NFAT cells, demonstrating considerable insensitivity to the mono/diester nature. Further modifications to the lipid chains were also found to engage Mincle; iso‐branched diesters (e.g., **54**) were also capable of Mincle‐dependent activation of BMDMs [224]. Modifications including OMe, epoxide or CHCH_2_OH at the β‐position of α‐branched lipids on trehalose were tolerated according to their comparable ability to activate hMincle NFAT cells, as well as mMincle NFAT cells, albeit more weakly [225]. Furthermore, an extra methylene group could be inserted between the sugar and the C6‐ester to generate compounds capable of signaling though hMincle and mMincle in NFAT cells (e.g., **55**) [226]. HEK cells indicated, for a panel of branched trehalose derivatives, that medium chain lengths (e.g., **56**) were potent activators of both mMincle and hMincle [227]. In addition, β‐Gentobiosyl diglyceride motifs (natural fungal Mincle ligands) [228] were also found to signal through mMincle and hMincle NFAT cells (e.g., **57** that bound directly to murine Mincle and stimulate cytokine production in BMDCs) [229]. α‐Glucosyldiglycerides bearing a variety of lipid chains were also shown to activate Mincle in reporter cell lines [230, 231, 232].

It was demonstrated that the incorporation of an aryl ring (**58**) at the distal terminus of the lipid at the 6‐position of glucose could still activate hMincle and mMincle NFAT cells [233]. This activation proceeded with a similar potency to glucose monocorynomycolate **59** (which was found to signal through Mincle, albeit more weakly than TDM, and not at all at lower concentrations) [223]. The weaker binding of **59** can be rationalized by the fact that, with only one sugar moiety, fewer interactions with the binding site on Mincle would be possible.

#### 5.2.4 Non‐Glucose‐Containing Mincle Ligands

The primary site is not limited to glucose; arabinose monomycolate **60** has also been identified as a Mincle ligand through activation of mMincle HEK reporter cells and BMDCs [216]. Furthermore, mannose monomycolate (**61**) and monocorynomycolate (**62**) were shown to bind hMincle‐Fc [131]. Glycerol monomycolate (GroMM) **63** was found to be a ligand of hMincle but not mMincle using NFAT reporter cells, Mincle‐deficient mice, and primary macrophage assays [234]. However, a study using HEK reporter cells reported the ability of GroMM to signal via hMincle and even more potently through mMincle [227]. One reason for this difference could be that the synthetic ligand tested on HEK reporter cells had a shorter lipid chain than the GroMM tested on NFAT reporter cells.

Nevertheless, it is worth noting that the literature concerning Mincle ligands contains a higher proportion of cell‐based assays and signaling studies than direct binding measurements such as nuclear NMR spectroscopy or SPR. NFAT and HEK Mincle reporter cells are commonly employed but have been shown to give conflicting results in some cases [131, 227]. The correlation between the ability to bind Mincle and inducing a biological effect is not trivial [235]. This disconnect between simply binding Mincle and being able to induce signaling has been ascribed to various phenomena that have been the subject of a recent review [21]. These factors include the overall Mincle structure (e.g., monomeric, dimeric, or heterodimeric with MCL) as well as the presentation of ligands (e.g., monomeric vs. multimeric or plate‐bound vs micellar). Furthermore, the results of reporter cell assays are not guaranteed to be translatable to primary cells, thus indicating the utility of additional in vitro test systems, such as primary wild‐type vs. Mincle‐deficient immune cell subsets [215, 227].

#### 5.2.5 Brartemicin and Related Mincle Ligands

Following the discovery that brartemicin **64** binds bMincle [236], but does not signal through Mincle [237], intense interest focused on developing analogs as potential adjuvants to increase the efficacy of vaccines. Both aromatic rings were required for high Mincle affinity; the removal of one ring led to a 30‐fold reduction in affinity [236]. Derivatization included: altering the stereochemistry of the trehalose linkage [236], modifying the substitution of the aromatic rings [236, 237, 238, 239, 240], triazole linked aryl rings bearing alkyl chains [241], biaryl derivatives [242], amide linkages [243, 244], sulfonamide linkages [243], and other specialized motifs [245]. A similar motif (aryl‐alkyl chain) was present in compound **65**, a modified substructure of a natural Mincle ligand PGL‐III [246]. Signaling in bMincle NFAT reporter cells was only detected if the C_6_ alkyl chain was present. Furthermore, the crystal structure (PDB: 8H4V) showed that terminal glucose occupied the primary Ca^2+^ site, engaging primarily via the 3‐OH and 4‐OH, as well as the 2‐OH hydrogen bonding to Arg182. The methyl group of C6‐OMe enables a hydrophobic interaction with Phe198. The alkyl chain was reported to induce the multimerization of Mincle, engaging the lipid binding groove of neighboring CTLDs. The rhamnose residues did not appear to interact with the protein.

#### 5.2.6 Endogenous Mincle Ligands and Derivatives

Mincle has been shown to also recognize endogenous ligands, including the spliceosome‐associated protein SAP130 [247] and cholesterol crystals/sulfate [248, 249]. Cholesterol crystals were able to activate hMincle, but not mMincle NFAT reporter cells [248]. SPR was later used to demonstrate that cholesterol sulfate could also be bound directly by hMincle, activate mMincle HEK reporter cells and induce proinflammatory cytokines in murine BMDCs [249]. Another endogenous compound, β‐glucosyl ceramide (β‐GlcCer) C24:1(15Z), but not lactosyl ceramide (LacCer) was shown to activate hMincle and mMincle NFAT reporter cells [250]. However, a later study using SPR showed that hMincle did bind LacCer, in addition to the gangliosides GM3, GD3 and GM1 [235]. The activation of hMincle and mMincle NFAT reporter cells by α‐GlcCer could be enhanced by the incorporation of a C2‐*exo*‐methylene group (**66**), possibly due to modulation of the conformation; a distorted chair would be expected [251]. Similar structural modifications have also been trialed for the β‐diastereomer. Linkage editing to replace the anomeric oxygen atom of β‐GlcCer with CH_2_ **67** or CHF (both stereoisomers) **68**/**69** led to more potent signaling in hMincle and mMincle NFAT reporter cells and increased cytokine production in BMDMs and BMDCs [252]. Similar analogs of α,β‐trehalose led to brartemicin‐like structures, of which the (*R*)‐CHF modification **70** showed improved signaling in hMincle and mMincle NFAT reporter cells compared to the unedited analog [253].

Despite the number of synthetic ligands discussed here, in many cases the distinct pathogen‐derived ligands of Mincle have not yet been identified. Pathogens that are recognized by Mincle include *Legionella pneumophila* [254], *Lactobacillus* spp. [74], *Orientia tsutsugamushi* [255], and helminths [256], among others. It could be possible that the identification of these motifs would reveal new ligand classes, upon which future synthetic campaigns could be based.

### 5.3 Dectin‐1

#### 5.3.1 Dectin‐1 Structure

Human Dectin‐1 can exist as one of eight possible isoforms (generated through alternative splicing), with isoforms A and B being the most common [257]. The difference between the two major isoforms is that only isoform A includes a stalk region; however, both have an extracellular CRD, transmembrane region, and cytoplasmic domain. Importantly, these two isoforms possess the same CRD and did not show any significant difference in their abilities to bind a variety of β‐glucans. Naturally derived ligands of Dectin‐1 include agonists such as curdlan (a microbial linear β‐1,3‐glucan) and schizophyllan (a fungal β‐1,3‐glucan with β‐1,6 side chains) [258]. Laminarin, an algal polysaccharide, is generally considered an antagonist of Dectin‐1; however, it has also been shown to act as an agonist in certain cases, even showing opposing effects on human and murine Dectin‐1 [259]. Laminarin is a linear β‐glucan consisting of β‐1,3 and β‐1,6 linkages (e.g., in a ratio of 2:1 in *Eisenia bicyclis*) [260]. It should also be noted that Dectin‐1 does not only bind glucose of β‐glucans but was also shown to bind core fucose on IgG [261].

The long loop of murine Dectin‐1 contains 35 amino acids; however, these residues are not capable of binding calcium in the way other CTLDs can [262]. Instead, Dectin‐1 can bind β‐glucans via a hydrophobic groove that includes Trp221, His223, and Tyr 228 [263]. Binding has been suggested to be at least partially mediated by ligand‐induced oligomerization, with laminarin having been shown to induce tetramer formation of murine Dectin‐1. The minimum glucan length that can be detected to bind to Dectin‐1 has been determined to be 10–11 glucose units long [264].

#### 5.3.2 Dectin‐1 Ligands

**FIGURE 8:**
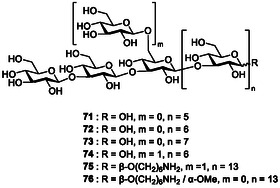
Structures of selected relevant synthetic Dectin‐1 ligands.^.^ Dectin‐1 ligands shown are based on linear and branched β‐(1,3)‐glucans (**71**‐**76**).

Synthetic ligands of Dectin‐1 have been based on typical naturally derived ligands: β‐glucans [258]. Expectedly, SPR revealed synthetic linear β‐(1,3)‐glucans were higher‐affinity binders of murine Dectin‐1 at longer lengths [265]. A linear 10‐mer **73** (Figure 8) (IC_50_   =   0.7 mM) vs. a linear 8‐mer **71** (IC_50_   =   1.1 mM) demonstrates this trend nicely. 1,6‐Branching was found to be beneficial in certain cases, e.g., linear 9‐mer **72** (IC_50_   =   2.6 mM) vs. branched **74** (IC_50_   =   29 μM). It should be noted that large natural polysaccharides are significantly better binders, e.g., laminarin (average M_r_   =   7.7 × 10^3^ gmol^−1^) is a particularly potent ligand (IC_50_   =   22 nM). Larger synthetic linear and branched β‐(1,3)‐glucans have been tested as ligands, with binding evaluated by NMR [266] and inhibition of Dectin‐1 binding to a schizophyllan‐functionalized surface [267, 268]. Branched **75** and linear **76** bound Dectin‐1 similarly, with a binding ∼10‐fold weaker than schizophyllan. With fewer β‐(1,3)‐glucose units, binding was much weaker, and the exchange of an anomeric β‐alkyl amino group for an α‐methyl glucoside in **76** abolished binding [268].

Owing to the large structures required for Dectin‐1 binding, synthetic access is a bottleneck to defined ligands, and many studies instead incorporate naturally derived material [265]. These natural glucans have been investigated, particularly in the context of Dectin‐1 targeting, to enhance immunogenicity during vaccination [269]. However, this carries risks of contamination, e.g., by lipopolysaccharides (LPS), and heterogeneity complicates structure–activity relationships (SARs) [270]. For the antagonist laminarin, a higher proportion of β‐(1,6) branches enhances its solubility, making the use of defined material even more attractive to ensure reproducible outcomes [260]. The evolution of synthetic methods, including automation, is a key driver in simplifying reproducible access to such complex structures. A solid‐phase automation platform was successfully employed to access β‐(1,3) linear and β‐(1,6) branched glucans, up to a branched 13‐mer [271, 272]. In terms of the generation of extremely large glycans, it is pertinent to highlight that an automated multiplicative approach enabled access to a polyarabinofuranose 1080‐mer [273]. With these synthetic advances, more detailed SARs could be determined for Dectin‐1, and more defined high‐affinity ligands could be identified, perhaps with therapeutic applications.

### 5.4 Langerin

#### 5.4.1 Langerin Structure

**FIGURE 9:**
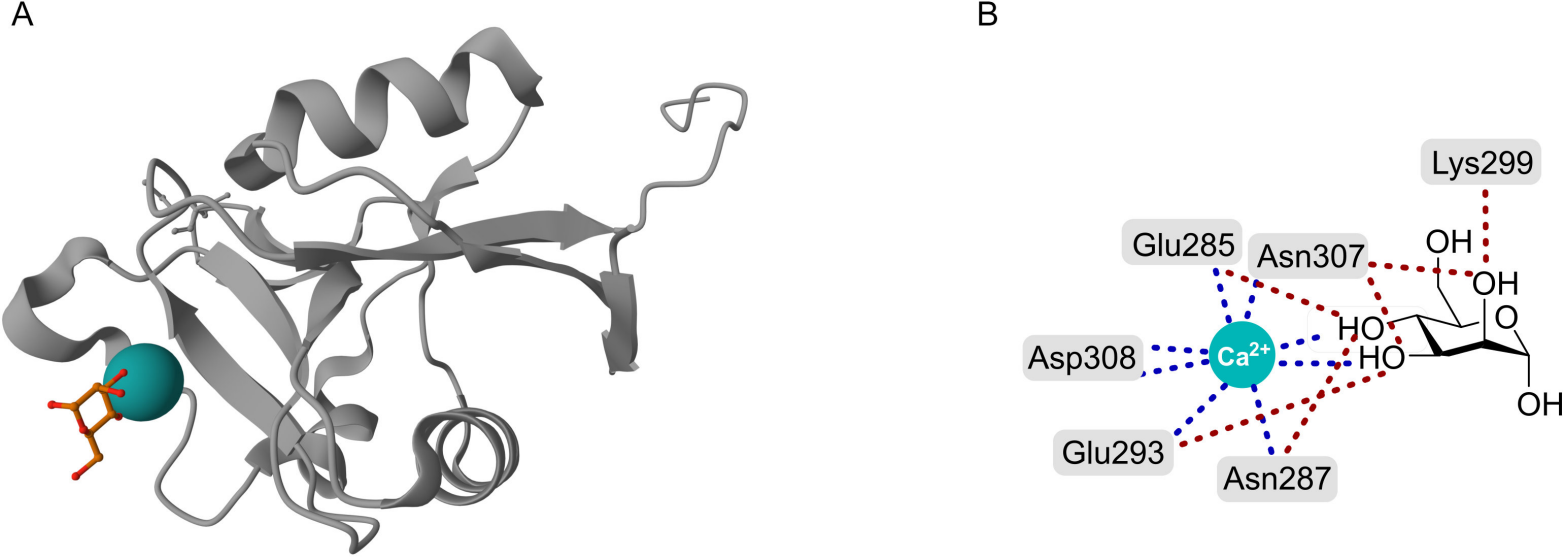
(A) Crystal structure of human langerin (gray) binding d‐mannose (orange/red) (PDB ID: 3P7G) [278], visualized with Mol*viewer [149]. Ca^2+^ ions (blue) have been enlarged for clarity. (B) Schematic representation of the key binding interactions. Shown in blue is the coordination of the protein and ligand to the Ca^2+^ ion. In red are other key ligand–protein interactions.

Langerin has been implicated in the binding of an array of pathogens, including fungi (e.g., *Candida*), bacteria (e.g., *Mycobacterium leprae*), and viruses (e.g., HIV and measles) [274]. With an EPN motif, langerin binds mannose‐containing, fucose‐containing and sulfated glycans, e.g., glycosaminoglycans (GAGs), as well as glucans [275, 276, 277]. Small carbohydrates like maltose or d‐mannose (PDB: 3P7G, Figure 9) can bind in a classical manner where the 3‐OH, 4‐OH vicinal diol coordinates Ca^2+^ and other residues (e.g., Asn307, Asp308, Glu285, Asn287, Glu293, and Lys299) stabilize the binding with a hydrogen‐bonding network [278]. For the GAG heparin (and synthetic heparin‐like molecules), two binding sites on langerin have been identified [279, 280]. Larger molecules bind (allosterically) independently of calcium to a positively charged groove between CRDs, whereas smaller molecules can bind to the classical Ca^2+^ binding site. Similar observations were also reported for chondroitin sulfate (CS) [281]. The presence of this Ca^2+^‐independent site is only present in multimerized CLRs, illustrating the importance of careful design of the CLR presentation during binding studies.

#### 5.4.2 Langerin Ligands

**FIGURE 10:**
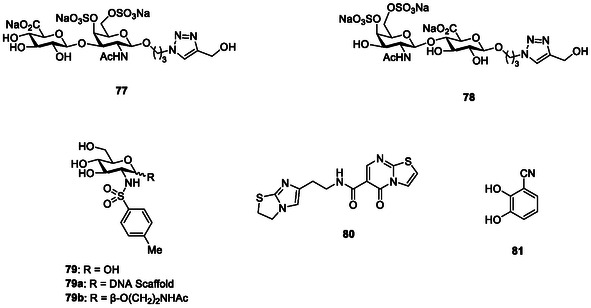
Langerin ligands including sulfated **77**‐**79** as well as non‐carbohydrate ligands that interact with an allosteric site (**80**) or the canonical site (**81**).

Chondroitin sulfate type‐E (CS‐E) disaccharides have been investigated as potential synthetic langerin ligands (Figure 10) [282]. With GlcA at the nonreducing end (**77**), there was a Ca^2+^‐dependent interaction. With the sulfated GalNAc at the nonreducing end (**78**), no binding was observed, presumably due to the lack of a vicinal 3‐OH, 4‐OH diol to coordinate the calcium. A variety of multivalent displays have been employed to improve binding to langerin, including glycopeptides, glycodendrimers, and glycoclusters [283, 284, 285, 286, 287]. Reflecting the importance of sulfated motifs [288, 289, 290], GlcNAc/heparin‐based glycomimetic **79** was identified as a promising langerin ligand (IC_50_   =   347 μM by SPR), for which binding could be increased ∼1150‐fold (IC_50_   =   300 nM) upon multimeric presentation on a DNA‐scaffold (**79a**) [291]. With an ethylamino linker, the binding mode of **79b** was proposed based on NMR and molecular docking [140]. The canonical Ca^2+^ interaction with 3‐OH and 4‐OH was supplemented by the aromatic ring engaging in a π–π interaction with Phe315. Furthermore, the sulfonamide linker at C2 could hydrogen bond with Asn307.

NMR‐enabled screening of a fragment library followed by ELISA and SPR revealed the thiazolopyrimidine motif, e.g., **80** (IC_50_   =   50 μM) to be an allosteric inhibitor of murine langerin [292]. The mechanism of this allosteric inhibition involved ligand binding in a region below the long loop, with a contribution from π−π interactions with Trp284. This binding consequently induced the release of Ca^2+^ from the protein [293, 294]. Additionally, other non‐carbohydrate inhibitors of langerin were found to engage with the canonical binding site, e.g., catechol **81** [295]. Rapid screening methods such as microarrays and NMR have already shown promise for identifying novel langerin binders. Innovations such as on‐chip synthesis of glycans are paving the way for expeditious ligand identification [296].

## 6 Summary and Outlook

The array of myeloid CLR ligands highlighted in this review demonstrates the intense effort that has been devoted to establishing the binding specificity of these lectins and developing high‐affinity mimetics, which may act as inhibitors or serve to activate signaling pathways. Despite the diversity of the CLRs discussed here, there are similarities seen in the signaling pathways as well as the binding specificities, which can be restrictive when aiming to develop selective CLR‐targeting therapeutics. Nevertheless, the potential of targeting myeloid CLRs for drug delivery, adjuvanticity, inflammation modulation, and viral inhibition is evident.

**FIGURE 11:**
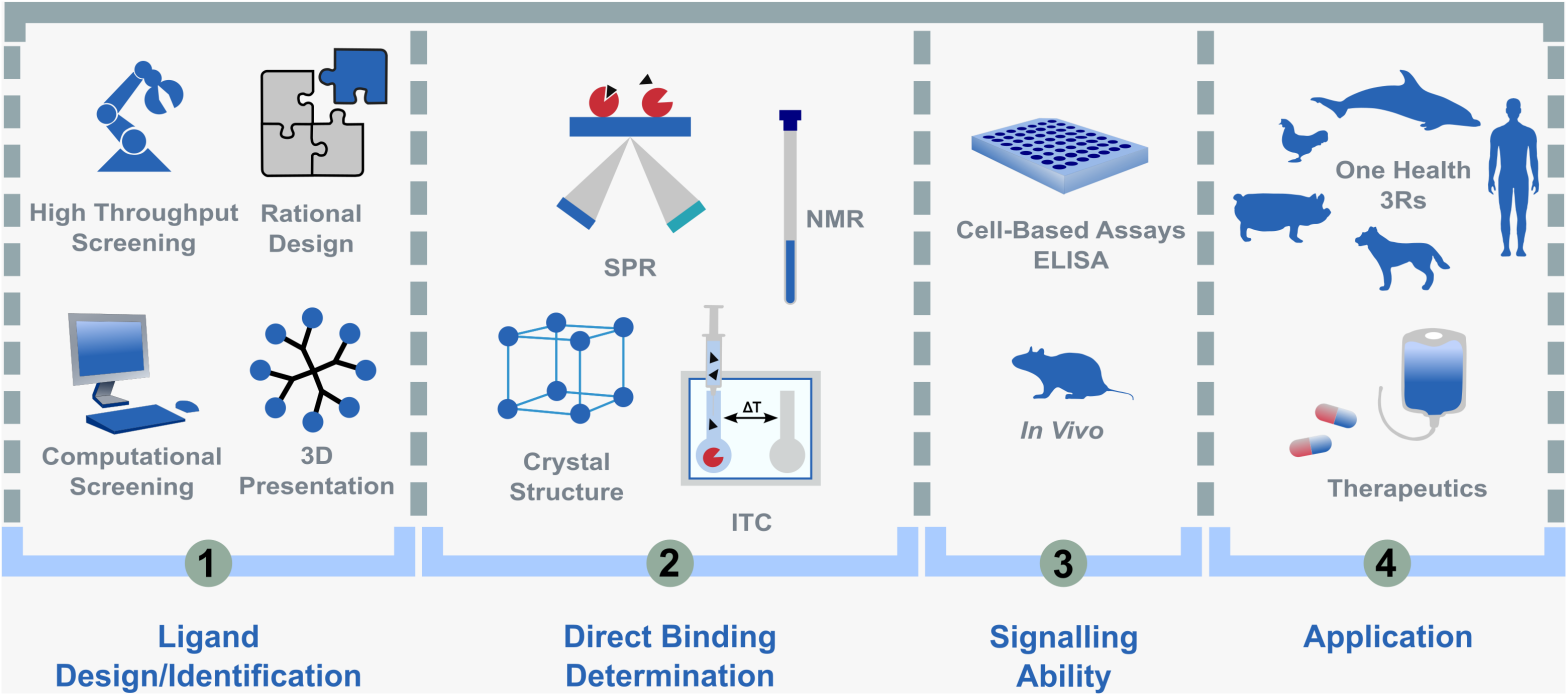
Overview of common techniques (not comprehensive) used to access ligands of CLRs. Initial identification of ligands benefits from automated and computational approaches. These designed ligands can then be validated by direct binding studies and then tested for their signaling ability in vitro and in vivo. The development of high‐affinity CLR ligands will drive their application in therapeutics and also increase understanding of CLR‐mediated processes that can be used to advance the themes of One Health and the 3Rs.

Synthetic ligand design based on existing SAR studies and crystal structures makes up a large proportion of existing publications [142]; however, this is increasingly being supplemented (or even replaced) with computational techniques (Figure 11) [200]. Even the lack of a crystal structure can now be overcome by using software like AlphaFold to predict protein structures for docking prospective ligands [297]. Experimental validation is still necessary, and most studies mentioned here used SPR, with some using NMR. Mincle is a slight exception, featuring a large number of reporter cell assays and fewer direct binding studies.

The gold standard for characterizing binding for both natural or synthetic ligands is the use of pure, defined compounds. However, the complex synthesis of glycans has hindered progress, although techniques such as automated glycan synthesis are emerging as solutions [298]. The complexity of comparing results across CLR studies is another limitation, which has been discussed in detail in the context of Mincle. Furthermore, the translation of initial binding studies to the modulation of innate responses in primary myeloid cells is an important consideration for which we currently do not have a thorough understanding, but rather empirical observations about which interactions are necessary, e.g., for Mincle [299]. Understanding factors affecting this, combined with binding requirements, could equip researchers with guidelines to better design novel agonists or antagonists.

With the idea that the field of CLR‐targeting therapeutics will grow in the future owing to the increasing number of reports on synthetic ligands, understanding interspecies differences is of utmost importance, particularly in the context of preclinical trials. The realization of the 3R principle in animal research (replacement, reduction, and refinement) can be aided by an understanding of the differences in CLR binding specificities and could lead to more efficient evaluations of potential therapeutics targeting CLRs [300]. As of writing, mostly human and murine (occasionally bovine) CLRs are tested whereas many common preclinical species are absent. This gap in our knowledge could be addressed by the implementation of a wider array of reporter cell assays that include common preclinical species, but also by the use of CLR libraries. For example, CLR‐Fc fusion protein libraries have been successfully employed for the detection of pathogen–CLR interactions for bacteria, viruses, fungi, and parasites [256, 301, 302, 303].

The study of CLRs from different species will also promote the One Health approach, taking into account the relationships within the natural world and contribute to the prevention and management of antimicrobial resistance and zoonoses [304]. The role of (targeting) CLRs in zoonoses, in particular, may represent a promising area of research in the aftermath of the SARS‐CoV‐2 pandemic [305].

With the identification of the vast number of ligands and common carbohydrate‐based pharmacophores for myeloid lectins described here, attention should focus on the 3D presentation of these compounds. Many examples in this review highlighted how a multivalent approach can enhance binding through avidity, though our understanding of exactly how to tailor a multivalent approach to a given lectin‐ligand pair is an area for development. The optimization of ligand displays will require careful investigation and is not a trivial task given the complexity of studying multivalent interactions and the large number of available scaffolds [306].

Taken together, the diversity and importance of CLR‐mediated processes in nature are clear, yet our understanding of such interactions, though impressive, is lacking. Synthetic ligands, aided by a panel of highly sophisticated analytical methods and multivalent scaffolds, are reconciling this disparity and will hopefully lead to the development of more CLR‐targeting therapeutics and bolster our understanding of host/pathogen interactions.

## Funding

This work was supported by Deutsche Forschungsgemeinschaft (LE 2498/10‐1 and LE 2498/14‐1 to B.L.).

## Conflicts of Interest

The authors declare no conflicts of interest.

## Data Availability

Data sharing not applicable to this article as no datasets were generated or analyzed during the current study.
